# Mao Jian Black Tea Ethanol Extract Alleviates Alcoholic Liver Injury in Mice via Regulation of the PI3K/Akt/NF-κB Signaling Pathway

**DOI:** 10.3390/foods14203492

**Published:** 2025-10-14

**Authors:** Lei Wu, Xiaomeng Guo, Yao Niu, Siyu Li, Shiyu Jiang, Xinyuan Wang, Yukang Gao, Shan Zhang, Litao Zhou, Lingdan Yang, Zian Gao, Yuqing Yang

**Affiliations:** 1 College of Life Sciences, Shanxi Agricultural University, Jinzhong 030801, China; ltwp159357@163.com (S.L.);; 2 Modern Research Center for Traditional Chinese Medicine, Shanxi University, Taiyuan 030006, China

**Keywords:** alcohol-induced liver injury, PI3K/Akt/NF-κB pathway, lipid metabolism, antioxidant, anti-inflammatory

## Abstract

This study investigates the protective effects and underlying mechanisms of Mao Jian Black tea ethanol extract (MJBT_EE) on a mouse model of acute alcohol-induced liver injury (ALI). The animal model was established using the NIAAA method, and C57BL/6 mice were divided into the following groups: negative control group (NC), model control group (MG), silibinin positive control group (SL, 54 mg/kg), and MJBT_EE high- and low-dose groups (40 mg/mL, 20 mg/mL). The results showed that, compared to the MG, MJBT_EE significantly reduced serum levels of ALT, AST, TC, TG, LDL-C, TBIL, ALP and inflammatory cytokines IL-6, TNF-α, and IL-1β (*p* < 0.01), while upregulating HDL-C (*p* < 0.01). It also enhanced the activity of hepatic antioxidant enzymes SOD and GSH (*p* < 0.01) and reduced MDA content (*p* < 0.01). Further histopathological examination of liver tissue revealed that MJBT_EE_H markedly alleviated hepatocellular hydropic degeneration, swelling, and steatosis. The mechanism of action of MJBT_EE_H primarily involved activation of the PI3K/Akt pathway and suppression of excessive p-NF-κB activation. These findings indicate that Maojian black tea ethanol extract exerts significant protective effects against alcohol-induced liver injury, potentially through improving lipid metabolism, reducing oxidative stress and inflammatory responses, and modulating the PI3K/Akt/NF-κB signaling pathway.

## 1. Introduction

The World Health Organization (WHO) estimates that approximately 2.3 billion people worldwide consume alcohol, with the majority concentrated in the United States, Europe, and the Western Pacific region [1]. The incidence of alcohol-related liver disease is particularly significant in European countries and is directly correlated with both the amount and duration of alcohol consumption [2]. Research indicates that alcohol-related liver disease progresses gradually. It initially manifests as the accumulation of fat in hepatocytes, a reversible stage known as fatty liver. Continued alcohol intake leads to the development of alcoholic hepatitis [3], with typical pathologies including fatty degeneration, necrosis, and acute inflammation. Ultimately, sustained alcohol exposure results in irreversible cirrhosis, exhibiting a “chicken-wire” fibrosis pattern (collagen deposition along terminal hepatic veins and sinusoids) and potentially leading to severe complications such as portal hypertension [4].

In strategies for the prevention and treatment of liver diseases, dietary natural active substances demonstrate unique value. These compounds (including tens of thousands of identified substances such as polyphenols, flavonoids, polysaccharides, and alkaloids) offer advantages such as low toxicity and multi-target effects, in addition to providing basic nutrition [5]. Mao Jian Tea (MJT) is derived from the northern part of Shanxi Province and is a medicinal tea made from *Dracocephalum rupestre* Hance of the Lamiaceae family. Preliminary studies have identified eriodictyol, eriodictyol-7-O-glucoside, luteolin, and luteolin-7-O-glucoside as its main active components [6]. Ordinary green tea contains about 3% caffeine [7], which can enhance the autonomous activity of the vagus nerve, release acetylcholine, and promote gastrointestinal peristalsis [8]. However, it can also cause euphoria and insomnia after consumption. In contrast, the caffeine content in MJT is only 0.495%, so it does not cause sleep disturbances [9]. Meanwhile, caffeine is also one of the main components contributing to the bitter taste of tea [10]. Existing research confirms that luteolin alleviates hepatic steatosis and injury by reversing ethanol-induced inhibition of AMPK and SREBP-1c phosphorylation [11]; luteolin-7-O-glucoside inhibits the proliferation of HepG2 liver cancer cells by inducing G2/M phase arrest through JNK activation [12]; both compounds also mitigate GalN/LPS-induced hepatotoxicity by modulating inflammatory mediators and phase II enzymes [13]. Eriodictyol alleviates LPS/D-GalN-induced ALI by activating the PI3K/AKT pathway, reducing oxidative stress and apoptosis [14]; it also improves non-alcoholic fatty liver disease by downregulating UBA52 to promote autophagy and activating the Nrf2/HO-1 pathway to inhibit oxidative stress [15]. These findings provide new directions for the prevention and treatment of alcohol-related liver disease, particularly highlighting the advantages of their multi-pathway and multi-target mechanisms over traditional drugs.

Based on this, we hypothesize that the flavonoid components in MJT may have protective potential against alcohol-induced liver injury. To test this hypothesis, in the preliminary stage of this study, we extracted the hydro extract and ethanol extract of Mao Jian black tea (MJBT). We found that the ethanol extract had a more typical effect on the ALI mouse model. Therefore, in this study, we evaluated its efficacy and explored its mechanism, aiming to provide a scientific basis for the in-depth development of this tea and its application in liver injury protection.

## 2. Materials and Methods

### 2.1. Extraction of the Ethanol Extract of MJBT

Two hundred grams of MJBT (Jiufeng Agricultural Products Processing Cooperative, Ningwu County, Shanxi, China) was weighed and extracted with 70% ethanol at a solid-to-liquid ratio of 1:20 g/mL for 60 min, yielding 76 g of fluid extract. The obtained fraction was concentrated using a rotary evaporator (200 mbar, 45 °C) and then dried in an oven (50 °C). After weighing, 28.5 g of the extract was obtained.

### 2.2. Animal Grouping, Administration, and Sample Collection

Thirty SPF-grade C57BL/6 mice (male, weighing 18 ± 22 g, purchased from Spelford Biotechnology Co., Ltd., Beijing, China) were acclimatized for one week. Clean water and animal maintenance feed were provided daily, with a 12 h light/dark cycle maintained. After that, 6 mice were randomly assigned to the negative control group (NC). The remaining 24 mice were used to establish the alcoholic liver injury model and randomly divided into four groups: the model group (MG), the silibinin-positive control group (SL, 54 mg/kg), the high-dose group of MJBT ethanol extract (MJBT_EE_H, 40 mg/mL), and the low-dose group (MJBT_EE_L, 20 mg/mL). 0.1% (or 1%) dimethyl sulfoxide (DMSO) was added to each group as a solubilizer. All random groupings were carried out after generating random numbers using the RAND function in Microsoft Excel (2021). Immediately after the grouping operation was completed, the generated random number sequence and the corresponding mouse numbers were recorded.

Modeling and Administration: The NC group was fed with the Lieber-DeCarli control liquid diet. The MG was fed with the alcohol-free Lieber-DeCarli liquid diet ( Nantong Trophic Animal Feed High-Tech Co., Ltd., Nantong, China) for the first 3 days of the first week; from days 4–7, they were fed with the Lieber-DeCarli liquid diet containing alcohol (the ethanol concentration gradually increased from 1% (*v*/*v*) to 4% (*v*/*v*)); from days 8–21, all mice except the NC group were fed a Lieber-DeCarli liquid diet containing 5% alcohol, while the SL and MJBT_EE groups began intragastric administration. On day 22, mice in the MG and administration groups were administered a 31.5% (*v*/*v*) alcohol solution by gavage at 5 g·kg^−1^, while the NC group was administered an equal dose of a 45% (*w*/*v*) maltodextrin solution. Nine hours later, the animals were anesthetized, and blood was collected from the abdominal aorta [16]. The blood was centrifuged at 2500 rpm for 10 min at −4 °C. Subsequently, liver tissue was collected from euthanized mice in each group. One part was placed in liquid nitrogen and stored at −80 °C for Oil Red O staining and Western blot analysis; another part was stored in 10% paraformaldehyde for H&E analysis.

All animal work was approved by the Experimental Animal Ethics Committee of Shanxi Agricultural University and was conducted in accordance with its regulations and guidelines as well as the ARRIVE guidelines. The animal experiment ethical review approval number is SXAU-EAW-2024M.SS.012025379.

### 2.3. Effects of MJBT_EE on Serum—Related Indicators

According to the operating procedures of the kits, the collected blood samples from mice were used to measure the relevant indicators of alanine aminotransferase (ALT, S03030, Rayto Life and Analytical Sciences Co., Ltd., Shenzhen, China), aspartate aminotransferase (AST,  S03040, Rayto Life and Analytical Sciences Co., Ltd., Shenzhen, China), high-density lipoprotein cholesterol (HDL-C, S03025, Rayto Life and Analytical Sciences Co., Ltd., Shenzhen, China), low-density lipoprotein cholesterol (LDL-C, S03029, Rayto Life and Analytical Sciences Co., Ltd., Shenzhen, China), triglyceride (TG, S03027, Rayto Life and Analytical Sciences Co., Ltd., Shenzhen, China), total cholesterol (TC, S03042, Rayto Life and Analytical Sciences Co., Ltd., Shenzhen, China), total bilirubin (TBIL, SP38661, Wuhan Saipei Biotechnology Co., Ltd., Wuhan, China), alkaline phosphatase (ALP, SP14295, Wuhan Saipei Biotechnology Co., Ltd., Wuhan, China), interleukin-6 (IL-6, D721022, Sangon Biotech Co., Ltd., Shanghai, China), tumor necrosis factor-α (TNF-α, D721217, Sangon Biotech Co., Ltd., Shanghai, China), and interleukin-1β (IL-1β, D721017, Sangon Biotech Co., Ltd., Shanghai, China).

### 2.4. Effects of MJBT_EE on Hepatic Oxidative Factor Indicators

Forty milligrams of liver tissue was weighed, 500 μL of extraction solution was added to an EP tube, and homogenization was performed in an ice bath. The homogenate was centrifuged at 10,000 r·min^−1^ for 10 min at 4 °C. The supernatant was collected and placed on ice for testing. The activity of superoxide dismutase (SOD, G4306, Wuhan Sevier Biotechnology Co., Ltd., Wuhan, China), glutathione (GSH, G4305, Wuhan Sevier Biotechnology Co., Ltd., Wuhan, China), and malondialdehyde (MDA, G4302, Wuhan Sevier Biotechnology Co., Ltd., Wuhan, China) in the liver were detected according to the respective kit instructions.

### 2.5. H&E Staining of Mouse Liver Tissue

The liver tissue, fixed in 4% paraformaldehyde, was paraffin-embedded and sectioned. The sections were then baked at 60 °C for 30 min and dewaxed in xylene. Next, they were rehydrated through a graded ethanol series (100% → 95% → 80% → 70%). After a wash step, hematoxylin staining was performed for 10 min, followed by rinsing under running water to achieve blueing. The sections were then counterstained with 0.5% eosin for a few seconds, dehydrated through a graded ethanol series (70% → 80% → 95% → 100%), and mounted with neutral balsam. Pathological changes were subsequently examined under an optical microscope.

### 2.6. Oil Red O Staining of Mouse Liver Tissue

Following cryosectioning of liver tissue, Oil Red O staining solution was applied to the sections for staining. The sections were then counterstained with hematoxylin and rinsed with water. Finally, the tissue was mounted with glycerin gelatin and observed under an optical microscope to assess changes in lipid droplet content. Using a semi-quantitative analysis method, the assessment is based on the percentage of the Oil Red O staining-positive area (red region) relative to the total area of the liver tissue section. The scoring criteria are as follows: Score 0, no steatosis or lipid vacuoles <5%; Score 1, mild steatosis (lipid vacuoles occupying 5–33% of the hepatic lobule area); Score 2, moderate steatosis (lipid vacuoles occupying 34–66% of the hepatic lobule area); Score 3, severe steatosis (lipid vacuoles occupying >66% of the hepatic lobule area) [17].

### 2.7. Western Blotting

Mouse liver tissue was weighed and ground with liquid nitrogen. RIPA lysis buffer containing 1% protease inhibitor and phosphatase inhibitor was added, and the mixture was left on ice for 30 min. It was then centrifuged (4 °C, 12,000 rpm, 10 min), and the supernatant was collected to obtain the protein stock solution. Protein quantification was performed using a BCA protein quantification kit. Proteins were separated using 10% sodium dodecyl sulfate-polyacrylamide gel electrophoresis (SDS-PAGE) gel electrophoresis and transferred to a polyvinylidene fluoride (PVDF) membrane. The membrane was blocked with protein-free rapid blocking buffer (G2052-500ML, Wuhan Servicebio Technology Co., Ltd., Wuhan, China) for 30 min. Primary antibodies were added, NF-κB (WL01980, Wanleibio Co., Ltd., Shenyang, China, 1:1000), p-NF-κB (WL02169, Wanleibio Co., Ltd., Shenyang, China, 1:1000), PI3K (bs-4160R, Biosynthesis Biotechnology Co., Ltd., Beijing, China, 1:1000), p-PI3K (4228, CST (Shanghai) Biological Reagents Co., Ltd., Shanghai, China, 1:1000), AKT (4691, CST (Shanghai) Biological Reagents Co., Ltd., Shanghai, China, 1:1000), and p-AKT (WLP001a, Wanleibio Co., Ltd., Shenyang, China, 1:1000), and incubated overnight at 4 °C. The membrane was washed three times with TBST buffer, for 10 min each time. HRP-labeled goat anti-rabbit IgG (H + L) secondary antibody (diluted 1:10,000) was added and incubated at room temperature for 2 h. The membrane was washed three times with TBST buffer and developed. It was placed on the exposure plate of a ChemiDoc MP all-in-one imaging system (Bio-Rad Laboratories, Inc., Hercules, CA, USA) for exposure and imaging in the darkroom. β-actin was used as the internal reference protein.

## 3. Data Analysis

Data are expressed as mean ± SEM. Statistical comparisons were performed using one-way analysis of variance (ANOVA) with SPSS version 26.0 (SPSS, Inc., Chicago, IL, USA) software. Visualization was conducted using GraphPad Prism version 7.0 (GraphPad Software, Inc., La Jolla, CA, USA).

## 4. Results

### 4.1. Preliminary Evaluation of the ALI Model

**Figure 1:**
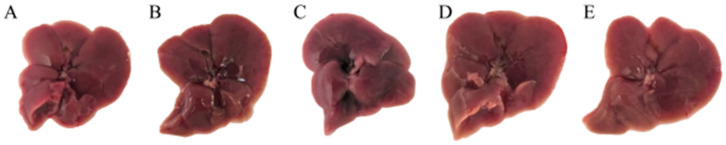
Effect of MJBT_EE treatment on the liver morphology of ALI mice. (**A**): Negative Control Group; (**B**): Model Group; (**C**): Silibinin-Positive Control Group; (**D**,**E**) represent the high- and low-dose groups of ethanol extract from MJBT, respectively.

Preliminary assessment of liver condition revealed that the livers in the NC and drug intervention groups were relatively smooth and glossy (Figure 1A,C–E). In contrast, the livers in the MG had a deeper, reddish-brown color compared to other groups, with a rough, granular surface, firm texture, and blunt, thickened edges (Figure 1B).

### 4.2. Effect of MJBT_EE on Liver Function in ALI Mice

**Figure 2:**
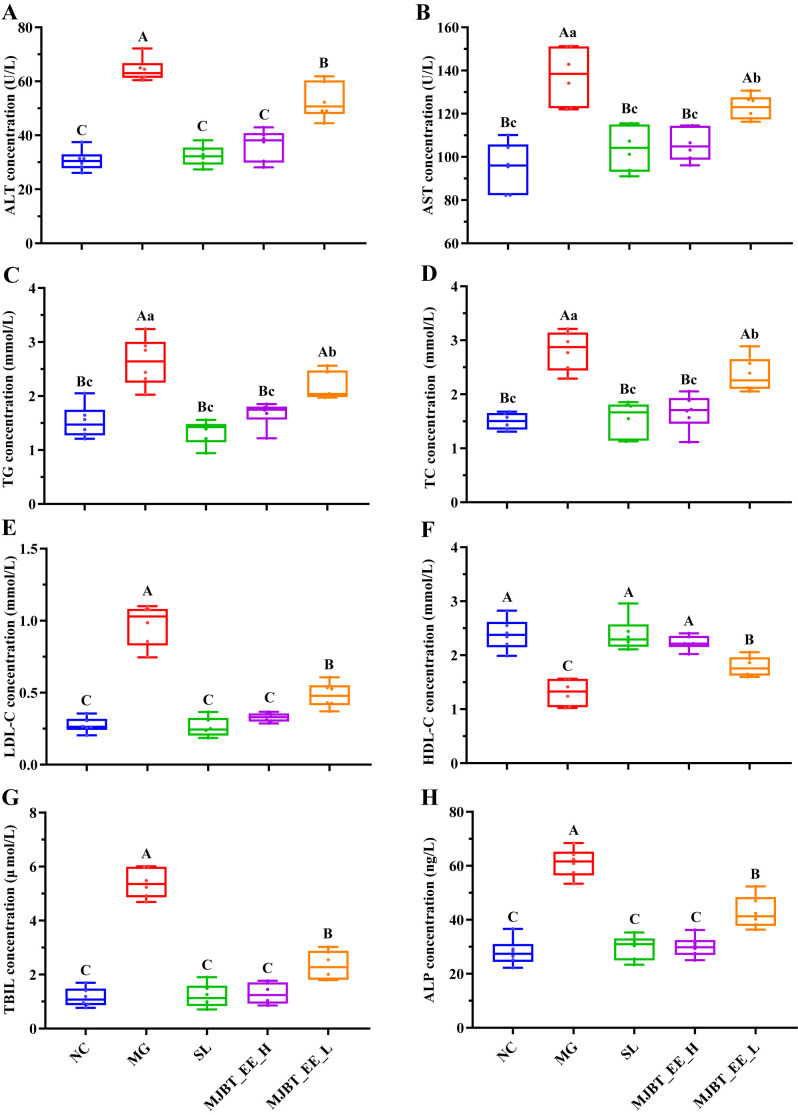
Effect of MJBT_EE treatment on ALT (**A**), AST (**B**), TG (**C**), TC (**D**), LDL-C (**E**), HDL-C (**F**), TBIL (**G**), and ALP (**H**) in ALI mice. Capital and lowercase letters above the bar indicate a significant difference at the 0.01 or 0.05 level, respectively. (*n* = 6 for each group). NC: Negative Control Group; MG: Model Group; SL: Silibinin Positive Control Group; MJBT_EE_H and MJBT_EE_L represent the high- and low-dose groups of ethanol extract from MJBT, respectively.

Alcoholic liver injury is often accompanied by abnormal serum lipid metabolism. Therefore, relevant indicators were measured. Compared to the NC, the MG showed significant increases in ALT, AST, TC, TG, LDL-C, TBIL and ALP (Figure 2A–E,G,H, *p* < 0.01). Treatment with SL, MJBT_EE_H, and MJBT_EE_L reversed these increases, but only the effect of MJBT_EE_H was on par with that of SL. Compared with the NC group, HDL-C was significantly upregulated in the MG (Figure 2F, *p* < 0.01). Similarly, SL, MJBT_EE_H, and MJBT_EE_L restored its levels, and only SL and MJBT_EE_H could regulate it back to NC levels.

### 4.3. Regulatory Effect of MJBT_EE on Hepatic Oxidative Factors in ALI Mice

**Figure 3:**
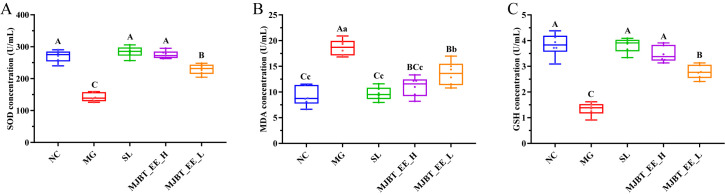
Effect of MJBT_EE treatment on SOD (**A**), MDA (**B**) and GSH (**C**) in ALI mice. Capital and lowercase letters above the bar indicate a significant difference at the 0.01 or 0.05 level, respectively. (*n* = 6 for each group). NC: Negative Control Group; MG: Model Group; SL: Silibinin-Positive Control Group; MJBT_EE_H and MJBT_EE_L represent the high- and low-dose groups of ethanol extract from MJBT, respectively.

Alcohol can lead to free radical production and the imbalance in hepatic oxidative stress response. Therefore, the extent of oxidative liver damage in mice was evaluated by measuring MDA, SOD, and GSH levels. Compared to the NC group, SOD and GSH were significantly decreased in the MG (Figure 3A,C, *p* < 0.01). Treatment with both SL and MJBT_EE_H restored them to NC levels, and their effects were superior to that of MJBT_EE_L (*p* < 0.01). Compared to the NC group, MDA was significantly upregulated in the MG (Figure 3B, *p* < 0.01). All intervention groups downregulated its levels (*p* < 0.01), and only SL and MJBT_EE_H restored them to NC levels.

### 4.4. Regulatory Effect of MJBT_EE on Inflammatory Factors in ALI Mice

**Figure 4:**
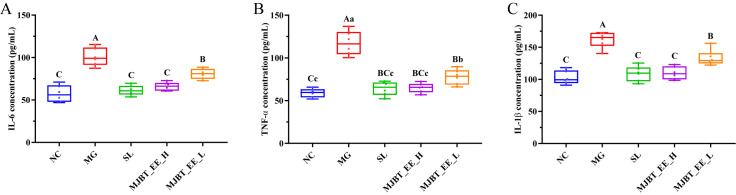
Effect of MJBT_EE treatment on IL-6 (**A**), TNF-α (**B**) and IL-β (**C**) in ALI mice. Capital and lowercase letters above the bar indicate a significant difference at the 0.01 or 0.05 level, respectively. (*n* = 6 for each group). NC: Negative Control Group; MG: Model Group; SL: Silibinin-Positive Control Group; MJBT_EE_H and MJBT_EE_L represent the high- and low-dose groups of ethanol extract from MJBT, respectively.

Inflammation is a core driver of the occurrence and progression of alcoholic liver disease. Selecting relevant inflammatory factors to evaluate the effect of MJBT_EE is crucial. The observed indicators, IL-6, TNF-α, and IL-1β, all showed significantly increased levels after successful modeling (Figure 4A–C, *p* < 0.01). SL, MJBT_EE_H, and MJBT_EE_L significantly reduced these levels (*p* < 0.01). The effects of SL and MJBT_EE_H were at the same therapeutic level (*p* > 0.01) and superior to that of MJBT_EE_L (*p* < 0.05).

### 4.5. Effects of MJBT_EE on Mouse Liver Observed by H&E Staining

**Figure 5:**
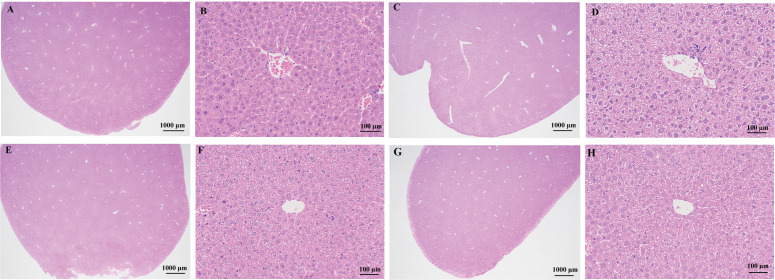
H&E staining to observe the effect of MJBT_EE_H on the liver tissues of ALI mice. (**A**) The liver tissues from normal mice; (**B**) The liver tissues from normal mice (200×); (**C**) The liver tissues of ALI mice; (**D**) The liver tissues of ALI mice (200×); (**E**) The liver tissues of ALI mice of SL treatment; (**F**) The liver tissues of ALI mice of SL treatment (200×); (**G**) The liver tissues of ALI mice of MJBT_EE_H treatment; (**H**). The liver tissues of ALI mice of MJBT_EE_H treatment (200×). (*n* = 6 for each group).

From the above results, MJBT_EE_H showed the best effect among the two concentration groups. Therefore, this concentration was selected for further investigation in subsequent experiments. To examine whether MJBT_EE has a reparative effect on damaged liver tissue, H&E staining was used to observe the tissue structure and cell morphology in each group. The results showed that in the NC, the liver tissue capsule was composed of dense connective tissue rich in elastic fibers with uniform thickness. The boundaries of the liver lobules were indistinct, and they were arranged regularly. The center of the lobule was the central vein, surrounded by radially arranged hepatocytes and hepatic sinusoids. The hepatocytes were round and plump; the hepatic plates were regularly and neatly arranged; the hepatic sinusoids showed no significant dilation or compression; the portal areas between adjacent lobules showed no significant abnormalities; and no significant inflammatory cell infiltration was observed (Figure 5A,B). After modeling, extensive hydropic degeneration of hepatocytes was observed in the MG, with cell swelling and pale-staining, loose cytoplasm; occasional focal lymphocyte infiltration was seen (Figure 5C,D). After drug treatment, the degree of hydropic degeneration in the SL was reduced, although cell swelling and pale-staining, loose cytoplasm remained; however, no significant inflammatory cell infiltration was observed (Figure 5E,F). The results for MJBT_EE_H were similar to those for SL, also showing mildly hydropic degenerated hepatocytes and no significant inflammatory cell infiltration (Figure 5G,H).

### 4.6. Effects of MJBT_EE on Mouse Liver Observed by Oil Red O Staining

**Figure 6:**
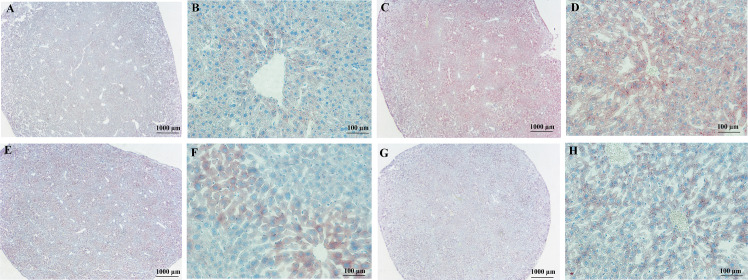
Oil Red O staining to observe the effect of MJBT_EE_H on the liver tissues of ALI mice. (**A**) The liver tissues from normal mice; (**B**) The liver tissues from normal mice (200×); (**C**) The liver tissues of ALI mice; (**D**) The liver tissues of ALI mice (200×); (**E**) The liver tissues of ALI mice of SL treatment; (**F**) The liver tissues of ALI mice of SL treatment (200×); (**G**) The liver tissues of ALI mice of MJBT_EE_H treatment; (**H**) The liver tissues of ALI mice of MJBT_EE_H treatment (200×). (*n* = 6 for each group).

In NC liver tissue stained with Oil Red O, lipids appeared red; small lipid droplets were widely observed in the cytoplasm of hepatocytes around the central veins (Score 0, Figure 6A,B). In the MG, lipids were stained red; small lipid droplets were widely observed in the cytoplasm of hepatocytes (Score 2, Figure 6C,D). In the SL, a small number of lipid droplets were visible in the cytoplasm, localized mainly around the central veins (Score 1, Figure 6E,F). After treatment with MJBT_EE_H, the lipid droplets in the hepatocyte cytoplasm were reduced compared to the MG (Score 1, Figure 6G,H).

### 4.7. Effect of MJBT_EE on the PI3K/Akt/NF-κB Signaling Pathway

**Figure 7:**
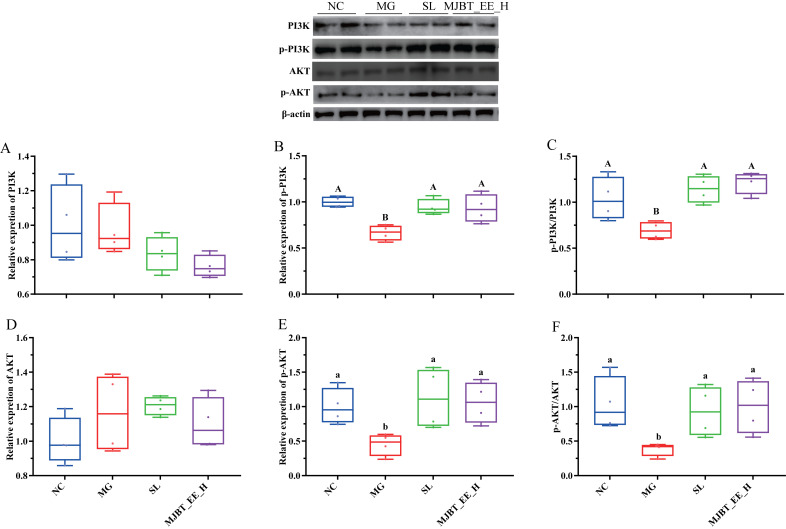
Effects of MJBT_EE_H treatment on the expression of key genes and proteins in the liver of ALI mice. (**A**) PI3K, (**B**) p-PI3K (**C**) p-PI3K/PI3K, (**D**) AKT, (**E**) p-AKT (**F**) p-AKT/AKT. Capital and lowercase letters above the bar indicate the difference significance at the 0.01 or 0.05 level, respectively (*n* = 4 for each group). NC: Negative Control Group; MG: Model Group; SL: Silibinin-Positive Control Group; MJBT_EE_H: the high-dose group of ethanol extract from MJBT.

The PI3K/Akt/NF-κB signaling pathway plays an extremely complex and critical role in the development and progression of ALI, coordinating the balance between hepatic inflammatory response and apoptosis [18]. The results showed that the expression levels of PI3K and AKT did not differ significantly among the groups (Figure 7A,D). However, their phosphorylated products, p-PI3K and p-AKT, were decreased in the MG (Figure 7B,E, *p* < 0.05). Similarly, the ratios of p-PI3K/PI3K and p-AKT/AKT also showed a decreasing trend (Figure 7C,F, *p* < 0.05). Treatment with both SL and MJBT_EE_H restored them to NC levels.

**Figure 8:**
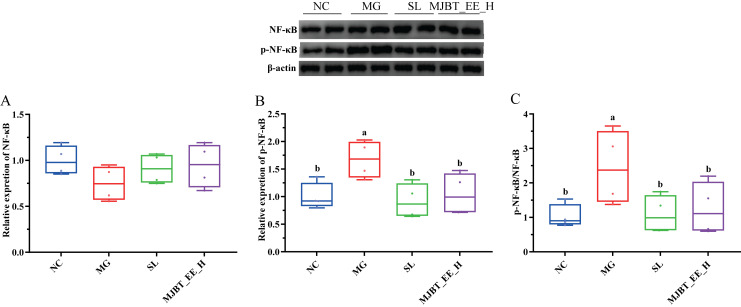
Effects of MJBT_EE_H treatment on the expression of key genes and proteins in the liver of ALI mice. (**A**) NF-κB, (**B**) p-NF-κB, (**C**) p-NF-κB/NF-κB. Lowercase letters above the bar indicate a significant difference at the 0.01 or 0.05 level, respectively. (*n* = 4 for each group). NC: Negative Control Group; MG: Model Group; SL: Silibinin-Positive Control Group; MJBT_EE_H: the high-dose group of ethanol extract from MJBT.

The expression level of NF-κB did not differ significantly among the groups (Figure 8A), but the expression level of its phosphorylated product p-NF-κB and the ratio of p-NF-κB/NF-κB were increased compared to the MG (Figure 8B,C, *p* < 0.05). Drug treatments also restored these to NC levels.

## 5. Discussion

This study, by constructing a mouse model of alcoholic liver injury and integrating multiple physiological, biochemical indicators and omics analysis, systematically evaluated the hepatoprotective effects of MJBT_EE for the first time and thoroughly explored its multi-faceted mechanisms of action. The results clearly demonstrate that MJBT_EE can effectively alleviate alcohol exposure-induced liver injury. Its mechanisms involve the regulation of antioxidant and anti-inflammatory activities, as well as related signaling pathways, providing a solid scientific basis for developing MJBT as a potential liver-protective functional food or adjunctive preparation.

Firstly, this study confirmed the significant effects of MJBT_EE in improving liver function indicators and lipid metabolism, with the high-dose group showing superior efficacy. This was primarily manifested by the significant reduction in serum ALT and AST activities following MJBT_EE treatment. The decline in these two transaminase levels not only suggests the maintenance of hepatocellular membrane structural integrity but also likely reflects an enhancement of mitochondrial function and antioxidant capacity in hepatocytes, thereby mitigating the combined damage from oxidative stress and inflammation triggered by ethanol metabolism [19,20]. Furthermore, the progression of alcoholic liver disease—from steatosis and alcoholic hepatitis to cirrhosis—leads to hepatocyte damage and impaired bile excretion. This affects both the uptake and conjugation of indirect bilirubin and the excretion of direct bilirubin. Total bilirubin level serves as a gold-standard indicator for assessing the liver’s overall capacity to process bilirubin. Extensive hepatocyte injury and cholestasis induced by alcoholic liver disease can both cause an elevation in total bilirubin. Thus, total bilirubin is a key and sensitive marker for evaluating the severity of liver dysfunction or injury. Chronic heavy alcohol consumption causes damage, inflammation, and necrosis of hepatocytes and biliary epithelial cells. This disrupts the fine biliary ductules within the liver, impairing normal bile production and excretion. When bile flow is obstructed, biliary components, including bile acids and bilirubin, regurgitate into the bloodstream. Concurrently, ALP located on the surface of cholangiocytes, is released into the systemic circulation, leading to elevated serum ALP levels [21]. In this study, significant increases in serum TBIL and ALP levels were observed in the ALI model group, while intervention with SL and MJBT_EE effectively reversed this trend. The reduction in TBIL and ALP by MJBT_EE further supports its ability to ameliorate ethanol-induced disorders in bilirubin metabolism and biliary function, consistent with the decreasing trends observed for ALT and AST. Regarding lipid metabolism, MJBT_EE exhibited significant regulatory effects: it markedly reduced TC, TG, and LDL-C levels, indicating its potential to synergistically ameliorate alcohol-induced lipid metabolism disorders through multiple pathways, thus inhibiting lipid accumulation and fatty acid synthesis and alleviating hepatic lipid metabolism dysfunction. Furthermore, the recovery in HDL-C levels further indicates that MJBT_EE may enhance reverse cholesterol transport function, facilitating the translocation and clearance of hepatic lipids to the peripheral circulation [22]. It is important to note that intrahepatic cholestasis and lipid metabolism disorders often interact in a mutually reinforcing manner in alcoholic liver disease. Impaired TBIL metabolism can affect hepatic lipoprotein assembly and secretion, while changes in ALP activity, as a membrane-bound enzyme, may also be related to alterations in the hepatocellular membrane lipid environment. Therefore, the regulatory effects of MJBT_EE on TBIL and ALP, together with its improvements in lipid metabolism parameters (TC, TG, LDL-C, HDL-C), may collectively constitute its overall mechanism in alleviating alcohol-induced liver injury, reflecting its multifaceted benefits in detoxification, excretion, and the reestablishment of lipid homeostasis in hepatocytes. This series of improvements in biochemical indicators was strongly supported at the histomorphological level—Oil Red O staining showed significantly reduced lipid droplet deposition in liver tissue, further confirming its positive role in alleviating alcoholic steatosis [23]. Existing literature indicates that lipid metabolism involves regulation by multiple transcription factors, such as peroxisome proliferator-activated receptor-α (PPAR-α), peroxisome proliferator-activated receptor-γ (PPAR-γ), sterol regulatory element-binding protein 1 (SREBP1), fatty acid synthetase (FASN), and carnitine palmitoyl transferase 1 (CPT1) [24,25]. Among these, PPAR-α can regulate mitochondrial fatty acid β-oxidation by controlling downstream target genes like CPT1, while PPAR-γ promotes cellular TG synthesis [26]. SREBP1 is a cholesterol regulatory element that controls the expression of upstream genes in lipid synthesis during fatty acid and TG biosynthesis; it also promotes FASN expression, increasing hepatic lipid synthesis [27]. FASN is a key metabolic enzyme in de novo fatty acid synthesis, regulating the body’s energy metabolism and balance [28]. Therefore, we speculate that the specific mechanisms may include, on one hand, inhibiting the activity of key genes in de novo lipid synthesis, such as SREBP-1c and FASN, to reduce hepatic de novo lipogenesis, and on the other hand, promoting fatty acid β-oxidation by activating the PPAR-α signaling pathway, thereby accelerating lipid consumption.

Oxidative stress is a core pathological link in the occurrence and development of alcoholic liver disease. During ethanol metabolism in the liver, large amounts of reactive oxygen species (ROS) are generated via pathways such as CYP2E1. The surge of ROS not only causes direct oxidative damage to hepatocytes but also leads to lipid peroxidation [29]. This process not only depletes key intracellular antioxidants such as GSH and reduces the activity of antioxidant enzymes like SOD but also causes the accumulation of lipid peroxidation end products (such as MDA), leading to structural damage in hepatocytes and triggering inflammatory responses [30,31]. The results of this study show that after MJBT_EE intervention, SOD activity and GSH levels in liver tissue significantly increased, while MDA content markedly decreased, indicating that the extract can effectively ameliorate alcohol-induced oxidative stress. It is worth noting that these antioxidant effects may be closely related to its rich flavonoid components (such as luteolin, eriodictyol, and their glycosides). These compounds not only possess direct free radical scavenging capabilities but may also activate endogenous antioxidant signaling pathways such as Nrf2/ARE, initiating the expression of Phase II detoxifying enzymes (e.g., SOD, nitric oxide synthase (NOS), catalase (CAT), glutathione peroxidase (GSH-Px)) and genes for antioxidant enzymes (e.g., Heme Oxygenase-1 (HO-1), γ-Glutamylcysteine Synthetase (γ-GCS), SOD, Quinone Oxidoreductase 1 (NQO1), as well as antioxidant proteins (e.g., bcl-2, Ferritin), thereby enhancing the liver’s overall resistance to oxidative stress. Therefore, MJBT_EE may not only neutralize ROS by providing exogenous antioxidants but also systematically restore the oxidant/antioxidant balance by regulating intracellular defense mechanisms, achieving multi-layered hepatoprotection.

The sustained activation of inflammatory responses is a key driver in the progression of alcoholic hepatitis to liver fibrosis and cirrhosis [19]. The PI3K/AKT signaling pathway is closely related to inflammatory responses. PI3K has various downstream effectors, with AKT being its direct target protein; activation of PI3K leads to phosphorylation of AKT. NF-κB is an important inflammatory transcription factor downstream of the PI3K/AKT signaling pathway. When the PI3K/AKT signaling pathway is downregulated, NF-κB protein is rapidly activated. Activated NF-κB translocates from the cytoplasm to the nucleus, promoting increased transcription of precursors like pro-Caspase-1. NLRP3 protein undergoes self-oligomerization and interacts with the adapter protein ASC, triggering a series of inflammatory cascades and leading to the massive release of downstream pro-inflammatory cytokines such as IL-6, TNF-α, and IL-1β [32]. Our research found that MJBT_EE_H can activate the PI3K/Akt signaling pathway. Concurrently, alcohol activated the NF-κB pathway (increased p-NF-κB levels), while MJBT_EE_H treatment effectively inhibited the phosphorylation and activation of NF-κB, thereby significantly reducing the levels of these inflammatory factors. Therefore, we speculate that the anti-inflammatory mechanism of MJBT_EE_H may lie in its active components activating the PI3K/Akt pathway, which then cross-talks with and suppresses the excessive activation of the NF-κB inflammatory signaling pathway. Specifically, previous studies have shown that flavonoid components such as luteolin, luteolin-7-O-glucoside, and eriodictyol can exert significant anti-inflammatory effects by regulating multiple key signaling pathways. Among these, NF-κB is regarded as a key target for the treatment of inflammatory responses. It is composed of p50, p52, p65 (Rel-A), c-Rel, and Rel-B proteins. In the NF-κB activated through the canonical pathway by pathological stimuli, the most common form is the p65:p50 heterodimer. The excessive increase in activated p65 and the subsequent transactivation of effector molecules are critical components in the pathogenesis of many chronic diseases, such as rheumatoid arthritis, inflammatory bowel disease, multiple sclerosis, and even neurodegenerative disorders. Therefore, the NF-κB p65 signaling pathway has consistently been a major focus in drug development [33]. Both luteolin and luteolin-7-O-glucoside can inhibit the phosphorylation of p65, but luteolin has a stronger inhibitory effect. Specifically, in the LPS-induced inflammation model, luteolin-7-O-glucoside has a weaker alleviating effect on the expression of iNOS and COX-2 compared with the potent inhibitory effect of luteolin [34]. The anti-inflammatory mechanism of luteolin also involves inhibiting the expression of NADPH oxidase 4 (NOX4) and reducing the production of reactive oxygen species (ROS), thereby effectively blocking the activation of NF-κB [35]. In addition, it can inhibit the phosphorylation of IκBα by IκB kinase (IKK), prevent the degradation of IκBα, and significantly reduce the expression of downstream pro-inflammatory factors such as TNF-α, IL-6, and IL-1β [36]. Meanwhile, luteolin can inhibit the phosphorylation of Akt and the increase in its mRNA level induced by TNF-α, suggesting that its anti-inflammatory effect is related to the regulation of the AKT pathway. Eriodictyol also shows the potential to regulate signaling pathways such as NF-κB and PI3K/Akt. It can inhibit the excessive activation of glial cells induced by LPS and regulate the expression of inflammatory cytokines by inhibiting the NF-κB and MAPK pathways [37]. In vitro experiments further indicate that eriodictyol can inhibit the production of NO, PGE2, MMP1, and MMP3 induced by IL-1β, and its mechanism is related to the reduction in the phosphorylation levels of PI3K, AKT, NF-κB p65, and IκBα [38].

Compared with existing hepatoprotective drugs, MJBT_EE exhibits a multi-target mechanism of action, which distinguishes it from many single-component polyphenol or flavonoid extracts. For example, silymarin and green tea extract mainly exert antioxidant effects by directly scavenging free radicals and activating the Nrf2 pathway, while MJBT_EE can simultaneously regulate lipid metabolism, oxidative stress, and inflammatory pathways. This comprehensive activity profile may confer advantages over hepatoprotective agents with limited mechanisms of action, but direct comparative studies are needed to validate its relative efficacy.

This study has several limitations that need to be addressed in subsequent research. Although we have identified potential active components, the specific contributions of luteolin, eriodictyol, and other components to the observed effects still need to be verified through compound isolation and purification. Additionally, the lack of dose–response relationship studies and pharmacokinetic analyses limits our understanding of the optimal dosing regimens and bioavailability of these compounds. The use of a short-term injury model also hinders the evaluation of the long-term hepatoprotective efficacy of MJBT_EE and its potential in the context of chronic liver diseases. Most importantly, its relevance to human physiology remains speculative until clinically validated. Future research should incorporate these elements to strengthen the evidence base for the therapeutic potential of MJBT_EE.

## 6. Conclusions

MJBT_EE exerts a protective effect against alcohol-induced liver injury through the synergistic actions of multiple pathways and targets. The mechanisms include (1) direct antioxidant activity alleviating oxidative stress; (2) regulation of the PI3K/Akt/NF-κB signaling pathway to suppress inflammatory responses; and (3) improvement of hepatic lipid metabolism disorders. In summary, this study systematically elucidates the protective effects of MJBT_EE against ALI through multiple pharmacological mechanisms, highlighting its potential for development as a functional food or adjunctive therapeutic agent. Further investigation into its dose–response relationships and pharmacokinetic analysis will be crucial for translating these findings into practical applications.

## Data Availability

The original contributions presented in the study are included in the article. Further inquiries can be directed towards the corresponding authors.

## Abbreviations

The following abbreviations are used in this manuscript:

MJBT_EE	Mao Jian Black tea ethanol extract
ALI	alcohol-induced liver injury
WHO	World Health Organization
MJT	Mao Jian Tea
MJBT	Mao Jian Black Tea
NC	Negative Control
MG	Model Group
SL	silibinin positive control group
MJBT_EE_H	high-dose MJBT alcoholic extract group
MJBT_EE_L	low-dose MJBT alcoholic extract group
DMSO	dimethyl sulfoxide
ALT	alanine aminotransferase
AST	aspartate aminotransferase
HDL-C	high-density lipoprotein cholesterol
LDL-C	low-density lipoprotein cholesterol
TG	triglyceride
TC	total cholesterol
TBIL	total bilirubin
ALP	alkaline phosphatase
IL-6	interleukin-6
TNF-α	tumor necrosis factor-α
SOD	superoxide dismutase
IL-1β	interleukin-1β
GSH	glutathione
MDA	malondialdehyde
SDS-PAGE	sodium dodecyl sulfate-polyacrylamide gel electrophoresis
PVDF	polyvinylidene fluoride
ANOVA	analysis of variance
PPAR-α	peroxisome proliferator-activated receptor-α
PPAR-γ	peroxisome proliferator-activated receptor-γ
SREBP1	sterol regulatory element-binding protein 1
FASN	fatty acid synthetase
CPT1	carnitine palmitoyl transferase 1
ROS	reactive oxygen species
NOS	nitric oxide synthase
CAT	catalase
GSH-Px	glutathione peroxidase
HO-1	Heme Oxygenase-1
γ-GCS	γ-Glutamylcysteine Synthetase
NQO1	Quinone Oxidoreductase 1
